# Performance of Thyme Oil@Na-Montmorillonite and Thyme Oil@Organo-Modified Montmorillonite Nanostructures on the Development of Melt-Extruded Poly-L-lactic Acid Antioxidant Active Packaging Films

**DOI:** 10.3390/molecules27041231

**Published:** 2022-02-11

**Authors:** Aris E. Giannakas, Constantinos E. Salmas, Areti Leontiou, Dimitrios Moschovas, Maria Baikousi, Eleni Kollia, Vasiliki Tsigkou, Anastasios Karakassides, Apostolos Avgeropoulos, Charalampos Proestos

**Affiliations:** 1 Department of Food Science and Technology, University of Patras, 30100 Agrinio, Greece; 2 Department of Material Science & Engineering, University of Ioannina, 45110 Ioannina, Greece; dmoschov@uoi.gr (D.M.); mbaikou@uoi.gr (M.B.); tasos.karakassides@gmail.com (A.K.); aavger@uoi.gr (A.A.); 3 Laboratory of Food Technology, Department of Business Administration of Agricultural and Food Enterprises, University of Patras, 30100 Agrinio, Greece; aleontiu@upatras.gr; 4 Laboratory of Food Chemistry, Department of Chemistry, National and Kapodistrian University of Athens Zografou, 15771 Athens, Greece; elenikollia@chem.uoa.gr (E.K.); vtsigkou@chem.uoa.gr (V.T.)

**Keywords:** thyme oil, sodium montmorillonite, poly-L-lactic acid, active packaging, antioxidant activity, antimicrobial activity

## Abstract

Today, the use of natural biodegradable materials in the production processes is more and more adopted by industry to achieve cyclic economy targets and to improve environmental and human health indexes. Active packaging is the latest trend for food preservation. In this work, nanostructures were prepared by incorporation of thyme oil with natural natrium-montmorillonite and organo-montmorillonite with two different techniques, direct impregnation and the green evaporation–adsorption process. Such nanostructures were mixed with poly-L-lactic-acid for the first time via an extrusion molding process to develop a new packaging film. Comparisons of morphological, mechanical, and other basic properties for food packaging were carried out via XRD, FTIR, TG, SEM/EDS, oxygen and water vapor permeation, and antimicrobial and antioxidant activity for the first time. Results showed that poly-L-lactic-acid could be modified with clays and essential oils to produce improved active packaging films. The final product exhibits food odor prevention characteristics and shelf-life extension capabilities, and it could be used for active packaging. The films based on OrgMt clay seems to be more promising, while the thyme oil addition improves their behavior as active packaging. The PLLA/3%TO@OrgMt and PLLA/5%TO@OrgMt films were qualified between the tested samples as the most promising materials for this purpose.

## 1. Introduction

Today, green and circular economies favor the development of nanocomposite packaging materials from renewable source-based biodegradable plastics or biopolymers [1]. Polylactides or poly(lactic acid) (PLA) is one of the most promising materials between the renewable source-based biodegradable plastics since it is thermoplastic, biodegradable, and biocompatible and exhibits high-strength, high-modulus, and good processability [2,3,4,5]. PLA is a linear aliphatic thermoplastic polyester produced either by the polycondensation of lactic acid or by the ring-opening polymerization of lactide. Lactide is a cyclic dimer prepared by the controlled depolymerization of lactic acid, which is obtained from the fermentation of renewable sugar feedstock, such as corn or sugar beets. Polymerization of a racemic of L-lactides leads to the synthesis of poly-L-lactide (PLLA). Since production cost has been lowered by new technologies and large-scale production, the application of PLA has been extended to materials for packaging applications [3,4,5,6].

Furthermore, one of the most recent applications of nanotechnology [7] in the food industry and food packaging sector is the development of polymer nanocomposite-based packaging films [8,9]. Among the nanomaterials used for polymer-based nanocomposites, clay-type nanomaterials are the most frequently used because they are naturally abundant low cost materials and because of the improved mechanical and barrier properties they add to the obtained polymer/clay nanocomposites [10]. More recently, clay-type nanomaterials have been proposed as an ideal nanocarrier for bioactive compounds, e.g., essential oils, for active food packaging films [8,11,12,13]. Such nanomaterials exhibit controlled released antimicrobial activity, antioxidant activity, and aroma properties. The obtained essential oils/nanoclay hybrids [14] can be added into polymer or biopolymer matrixes, providing active packaging films and/or coatings with potential controllable antioxidant and/or antimicrobial activity [15]. The term active packaging refers to packaging technologies which are used with foods, pharmaceuticals, and other products. The aim of the use of such technologies is to extend products shelf-life, to monitor their freshness and quality, and to increase their safety and accessibility by consumers [16].

Rhim et al. [17] prepared via a solution casting method PLA-based nanocomposite films with different types of nanoclays, such as commercial Cloisite Na^+^, Cloisite 30B, and Cloisite 20A. Cloisite 20A was the most effective to improve the water vapor barrier property and Cloisite 30B to improve the antibacterial properties. Recently, Ramos et al. [18] prepared PLA/thymol/nanoclay nanocomposite film via a melt-extruded method. They added 2.5 wt% and 5 wt% of a commercial nanoclay with code name Dellite^®^43B (D43B) and 8 wt% of thymol in PLA. The results from this study indicated that the diffusion of thymol through the PLA matrix was influenced by the presence of the nanoclay. Villegas et al. [6] prepared intercalated PLA/organoclay/nanocomposites film via melt extrusion and impregnated with thymol and cinnamaldehyde through supercritical impregnation using carbon dioxide.

Recently, PLA was incorporated with Na-montmorillonite (NaMt) and commercial organomodified montmorillonite (OrgMt) via two methods, the direct method and the masterbatch method to produce active packaging films. Comparison and evaluation of the different materials and the different producing processes were carried out via mechanical properties and structure characterization measurements and the results reported in [19]. In this work a direct impregnation and a novel green evaporation–adsorption method were used to modify Na-montmorillonite (NaMt) and commercial organomodified montmorillonite (OrgMt) with an essential oil, thyme oil (TO), to produce nanostructures similar to others reported in [20]. In the past, these obtained ΤO@NaMt and ΤO@OrgMt hybrid nanostructures were connected successfully with chitosan [21], Low-Density Polyethylene [12], and polystyrene [22] matrixes. The novelty of this work is that these new nanohybrid materials, i.e., the TO@NaMt and the ΤO@OrgMt, which were prepared via a classic method and via a novel evaporation–adsorption process, were used to develop novel poly-L-Lactic acid (PLLA)/thyme oil/montmorillonite nanocomposite active films via an extrusion molding process. The obtained PLLA/TO@NaMt and PLLA/TO@OrgMt films were characterized by XRD analysis, FTIR spectroscopy, SEM images, and TG experiments. Moreover, to evaluate the potential use of such active films in the food packaging industry, tensile properties, water, and oxygen barrier properties, as well as the antioxidant and antimicrobial activity of all the obtained PLLA/TO@NaMt and PLLA/TO@OrgMt films, were measured.

## 2. Results

### 2.1. XRD

Table 1 lists the code names of all samples and the used amounts of PLLA, NaMt, OrgMt, TO@NaMt, and TO@OrgMt.

**Figure 1:**
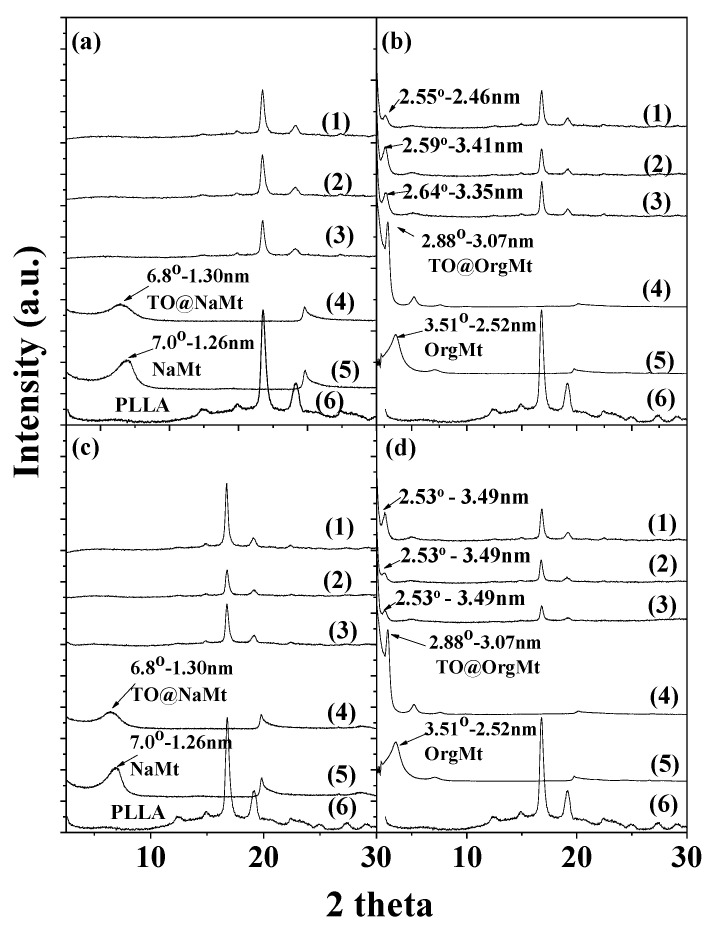
XRD plots of (**a**) PLLA/1NaMt (1), PLLA/3NaMt (2), PLLA/5NaMt (3), TO@NaMt hybrid nanostructure (4), NaMt as received (5), and PLLA (6); (**b**) PLLA/1OrgMt (1), PLLA/3OrgMt (2), PLLA/5OrgMt (3), TO@OrgMt hybrid nanostructure (4), OrgMt as received (5), and PLLA (6); (**c**) PLLA/1TO@NaMt (1), PLLA/3TO@NaMt (2), PLLA/5TO@NaMt (3), TO@NaMt hybrid nanostructure (4) NaMt as received (5), and PLLA (6); (**d**) PLLA/1TO@OrgMt (1), PLLA/3TO@OrgMt (2), PLLA/5TO@OrgMt (3), TO@OrgMt hybrid nanostructure (4), OrgMt as received (5), and PLLA (6).

Figure 1 presents the XRD plots of PLLA/NaMt (Figure 1a), PLLA/OrgMt (Figure 1b), PLLA/TO@NaMt (Figure 1c), and PLLA/TO@OrgMt (Figure 1d) nanocomposite films. XRD plots of pure PLLA, NaMt, OrgMt as received, and TO@NaMt and TO@OrgMt nanostructures are also included to compare with the obtained films.

PLLA shows its most intense diffraction peaks at 2θ values of 16.6 and 18.96 degrees, which is in agreement with previous reports [23,24]. The intension of such peaks decreased in all XRD plots of PLLA/NaMt, PLLA/TO@NaMt, PLLA/OrgMt, and PLLA/TO@OrgMT nanocomposites. This fact indicates the interplay between PLLA molecules and nanoclay platelets. The decrease of intense of the characteristic PLLA peaks is higher for OrgMt- and TO@OrgMt-based nanocomposites than for NaMt- and TO@NaMt-based nanocomposites.

It is obvious from Figure 1a,c that the major diffraction peak of NaMt clay is at around 2theta = 7.0°. The TO@NaMt modified nanostructure exhibits a broader peak at 2theta = 6.8°. According to previous reports [14,20], TO molecules were encapsulated on the external surface of NaMt layers, causing a partial exfoliation of clay platelets. The small shift of 2theta angle from 7.0 to 6.8° occurs probably due to the insertion of water molecules in the NaMt’s interlayer space. It is also obvious from Figure 1a,c that the blending of both NaMt clay and TO@NaMt nanostructure with PLLA chains resulted to XRD plots with a very broad peak at 2theta around 4.8 and 5.0 degrees. Thus, it seems that PLLA/NaMt and PLLA/TO@NaMt nanocomposites favored the formation of an exfoliated nanocomposite structure [12].

In Figure 1b,d, the basal space of OrgMt clay is observed at 2theta = 3.50°. TO@OrgMt hybrid nanostructure exhibits a clear shift at 2theta = 2.88° degrees. This result is in accordance with previous report [20]. In this report, it was shown [20] that TO molecules encapsulated in the internal space of OrgMt clay platelets. The basal space peak of PLLA/1OrgMt, PLLA/3OrgMt, and PLLA/5OrgMt film was at 2theta, 2.55°, 2.59°, and 2.64°, respectively, while the basal space peak of all PLLA/1O@OrgMt, PLLA/3TO@OrgMt, and PLLA/5TO@OrgMt films was at around 2theta = 2.53°. These results indicated the formation of an intercalated nanostructure in all cases, and the boost that TO molecules provided to PLLA chains to insert in the OrgMt clay interlayer space.

### 2.2. FTIR

**Figure 2:**
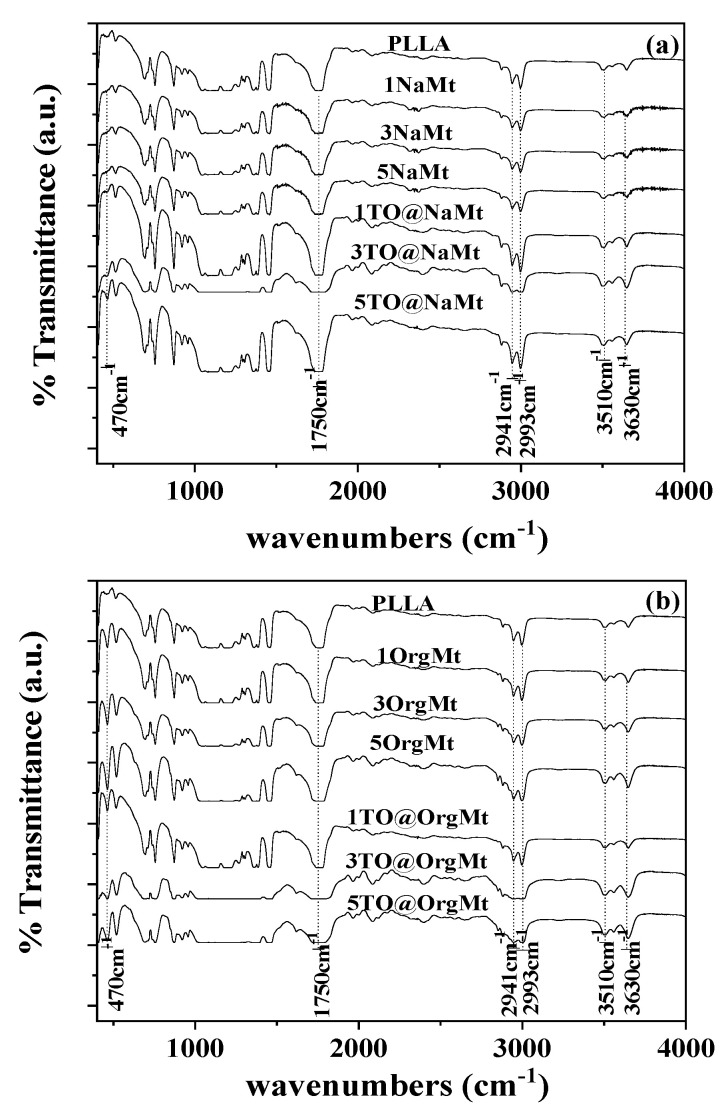
FTIR plots of (**a**) pure PLLA, PLLA/xNaMt, and PLLA/xTO@NaMt nanocomposites; (**b**) pure PLLA, PLLA/xOrgMt, and PLLA/xTO@OrgMt nanocomposites.

Figure 2 presents the FTIR spectra of all PLLA/xNaMt, PLLA/xTO@NaMt (Figure 2a), and PLLA/xOrgMt, PLLA/xTO@OrgMt, (Figure 2b) nanocomposite films as well as FTIR spectra of “blank” PLLA film for comparison. In all spectra, the main characteristic peaks of both PLLA are observed. As it is denoted (see dot lines in Figure 2a,b), at 3510 cm^−1^ the OH stretch band, at 2941 and 2993 cm^−1^ the CH and CH3 stretching bands, and at 1750 cm^−1^ the characteristic band of the CO ester group of PLLA chain is observed [25].

In such polymer/clay nanocomposite films, the interplay with clay platelets is indicated by the new bands at 3630, 918, 660, and 470 cm^−1^, which are accredited to O-H stretching of structural hydroxyl groups, Al-O stretching, Mg-O bond, and Si-O stretching of the tetrahedral silica layers of modified clays, respectively [26,27]. In the case of both NaMt- and OrgMt-based nanocomposites, the bands at 3630, and 470 cm^−1^ of Al-O and Si-O stretchings are most identified (see dot line in Figure 2a,b) and imply the interplay between clay platelets and PLLA chains. With a more careful look at Figure 2 (see the band at 470 cm^−1^), it is observed a higher interplay for OrgMt-based than NaMt-based samples. In advance, this interplay is higher for TO@NaMt and TO@OrgMt films as compared to NaMt and OrgMt films, correspondingly. This probably occurs due to the hydrophobic nature of the PLLA, which is matched with the hydrophobic nature of the TO. The absence of FTIR peaks, which indicate the existence of TO molecules and its derivatives, implies that such molecules are inside the polymer matrix and not on the surface of it. Moreover, the TO amount is too low compared to that of the PLLA and the clay.

### 2.3. TG–DTA

**Figure 3:**
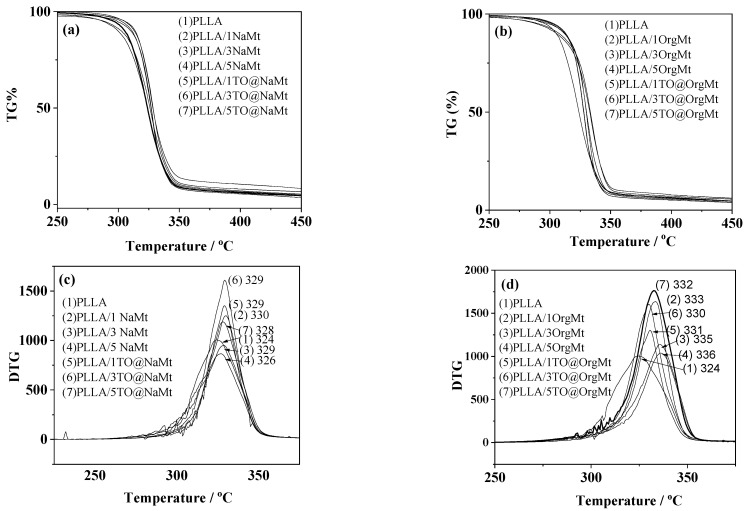
TG plots of (**a**) pure PLLA, PLLA/xNaMt, and PLLA/xTO@NaMt nanocomposites, and (**b**) pure PLLA, PLLA/xOrgMt, and PLLA/xTO@OrgMt nanocomposites. DTG plots of (**c**) pure PLLA, PLLA/xNaMt, and PLLA/xTO@NaMt nanocomposites, and (**d**) pure PLLA, PLLA/xOrgMt, and PLLA/xTO@OrgMt nanocomposites.

In Figure 3, the TG plots of pure PLLA, “blank” PLLA/NaMt, and PLLA/OrgMt samples as well as all obtained PLLA/TO@NaMt and PLLA/TO@OrgMt nanocomposite films are presented. For all samples, the degradation step starts at around 300–320 °C and ends at 350–400 °C.

The temperature value where the degradation process starts is indicative for the thermal strength of such PLLA/nanoclay nanocomposite films. DTG plots for each sample are depicted in Figure 3, and the calculated degradation temperature for each sample is listed. In the case of NaMt-based samples, the addition of extra NaMt caused a slightly increase to the degradation temperature of samples. Moreover, the addition of TO caused a slight decrease to the degradation temperature compared with samples without TO. The addition of extra OrgMt to the OrgMt-based nanocomposites showed a higher increase of the degradation temperature, compared to respective of the NaMt-based samples. The addition of TO caused a slight increase to the degradation temperature of PLLA/1TO@OrgMt as compared to the respective of the PLLA/1OrgMt sample but a slight decrease to the degradation temperature of the PLLA/3TO@OrgMt and PLLA/5TO@OrgMt samples as compared to the respective of the PLLA/3OrgMt and PLLA/5OrgMt samples. The higher increase in thermal stability obtained for OrgMt-based samples as compared to NaMt-based samples is expected following XRD results where intercalated nanocomposite structures were obtained for all OrgMt-based samples, while exfoliated nanocomposite structures were obtained for all NaMt-based samples. The slight decrease of thermal stability of samples containing TO as compared to the samples that do not contain TO is indicative of the plasticization effect caused by the TO addition.

### 2.4. Tensile Properties

Young’s modulus as well as tensile strength and elongation at break values of the tested films are tabulated in Table 2. It is obvious that the addition of NaMt in all cases decrease stiffness, strength, and elongation at break values of PLLA. Addition of modified TO@NaMt nanostructure leads to higher stiffness, strength, and elongation at break values of obtained PLLA/TO@NaMt films compared to the relevant PLLA/NaMt films, but only PLLA/3TO@NaMt films exhibit a little higher stiffness, strength, and elongation at brake values compared to the values exhibited by pure PLLA film. In contrast, addition of OrgMt in the PLLA matrix seems to be more effective.

Because of this, the PLLA3OrgMt sample showed higher stiffness, strength, and elongation at break values than pure PLLA. Moreover, addition of TO@OrgMt nanostructure leads to the even higher stiffness, strength, and elongation at break values. In other words, it seems that the OrgMt nanostructure exhibits better interplay with the PLLA matrix than the NaMt nanostructure. This result is in accordance with [17]. The addition of TO boosts the dispersion of both NaMt and OrgMt nanostructures in the PLLA matrix. Optimum interplay is observed for the PLLA/TO@OrgMt system. The highest stiffness, strength, and elongation at break values were observed for PLLA/3TO@OrgMt active film.

### 2.5. SEM/EDS Results

The surface morphology of the pure PLLA and PLLA/clay nanocomposite films was investigated using a SEM instrument equipped with an EDS detector. EDS mapping analysis from different areas of the studied nanocomposite films was recorded, and the results confirmed that the clays were homogeneously dispersed in the polymer matrix.

**Figure 4:**
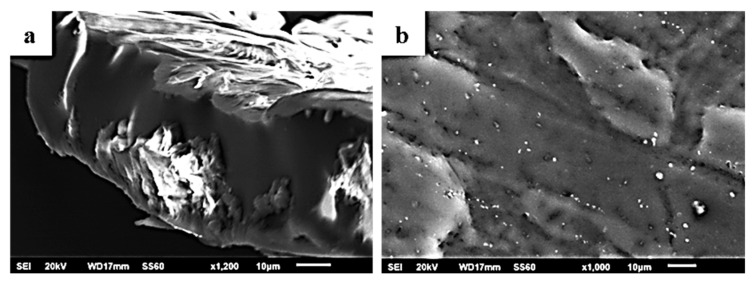
SEM images of the pure PLLA film: (**a**) cross-section and (**b**) surface.

**Figure 5:**
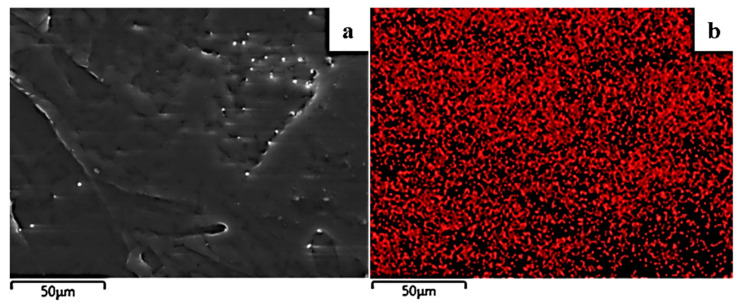
Secondary electron SEM image (**a**) and respective EDS compositional mapping of carbon (**b**) for the pure PLLA film.

**Figure 6:**
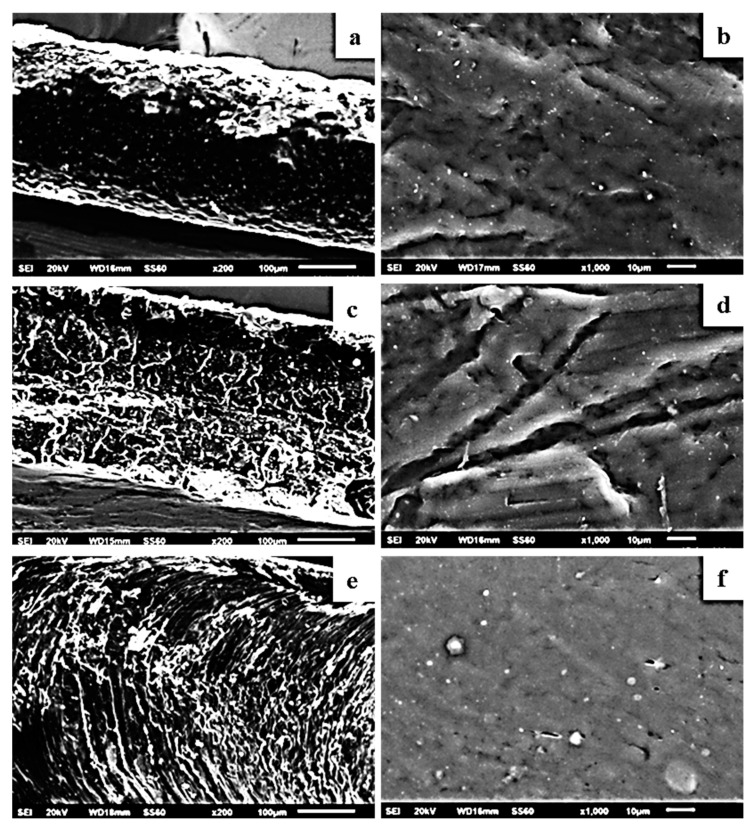
SEM images of cross-section and surface for the films: (**a**,**b**) PLLA/1%TO@NaMt, (**c**,**d**) PLLA/3%TO@NaMt, and (**e**,**f**) PLLA/5%TO@NaMt.

**Figure 7:**
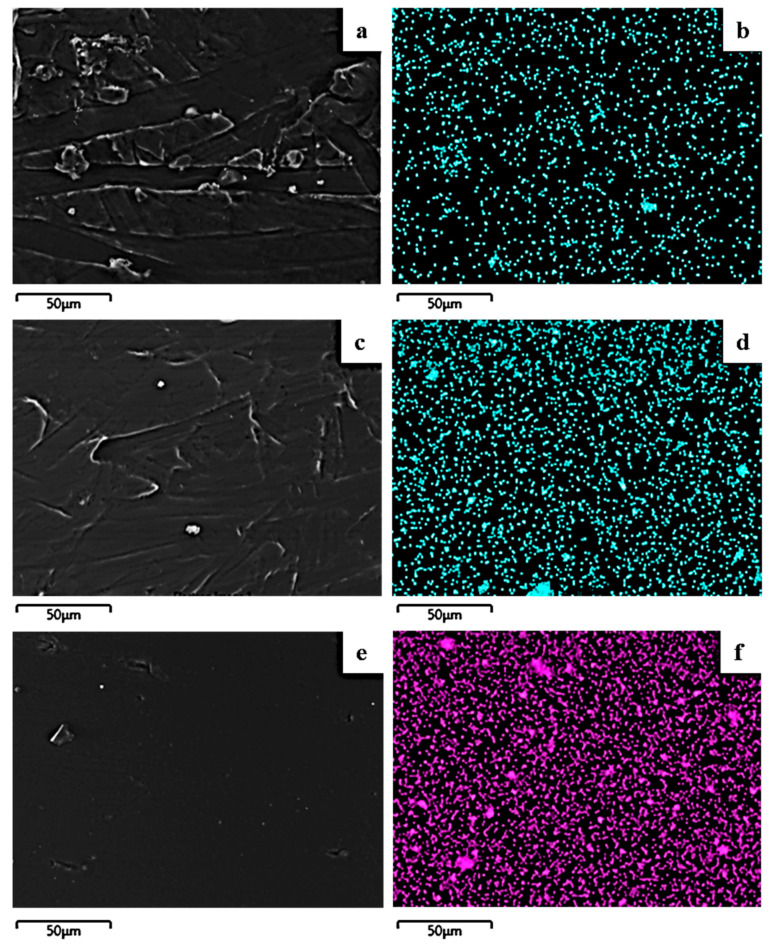
Secondary electron SEM images and respective EDS compositional mapping of silicon for the films: (**a**,**b**) PLLA/1%TO@NaMt, (**c**,**d**) PLLA/3%TO@NaMt, and (**e**,**f**) PLLA/5%TO@NaMt.

**Figure 8:**
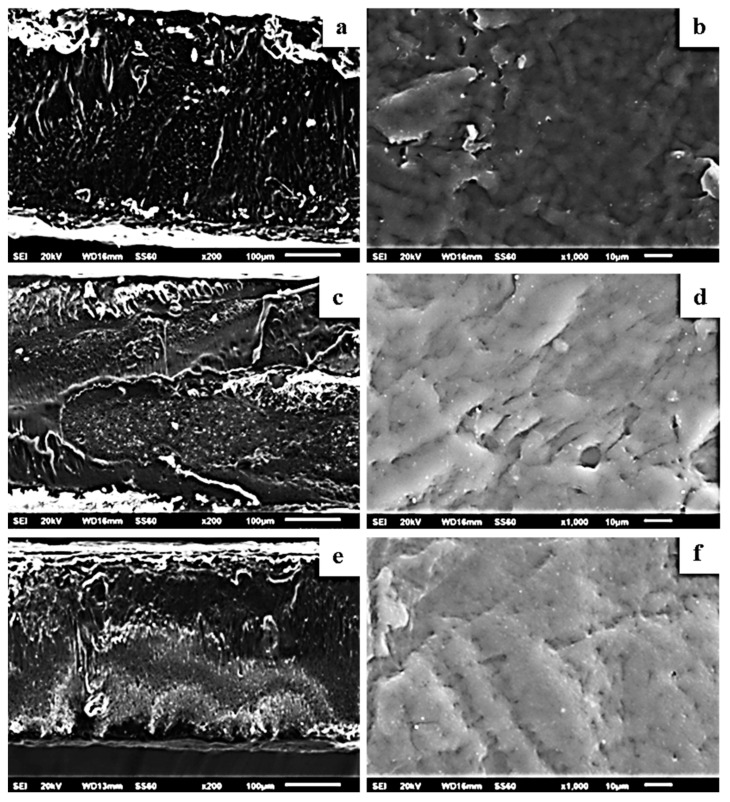
SEM images for the films: (**a**,**b**) PLLA/1%TO@OrgMt, (**c**,**d**) PLLA/3%TO@OrgMt, and (**e**,**f**) PLLA/5%TO@OrgMt.

The image in Figure 4 shows the expected smooth morphology of the neat polymer, and in Figure 5 the EDS spectra certify the existence of carbon. Surface and relative cross-section images of PLLA/1TO@NaMt, PLLA/3TO@NaMt, and PLLA/5TO@NaMt are presented in Figure 6. It is obvious from Figure 7a,c,e that the increase of clay content in the nanocomposite materials increases the aggregation degree. This fact can be identified by SEM using a secondary electron signal. EDS was then used to map specific elements of the clay nanostructure and identify especially the presence of silicon. As it is observed in Figure 8, clay agglomeration took place, and the increase in clay concentration led to increased silicon signal.

**Figure 9:**
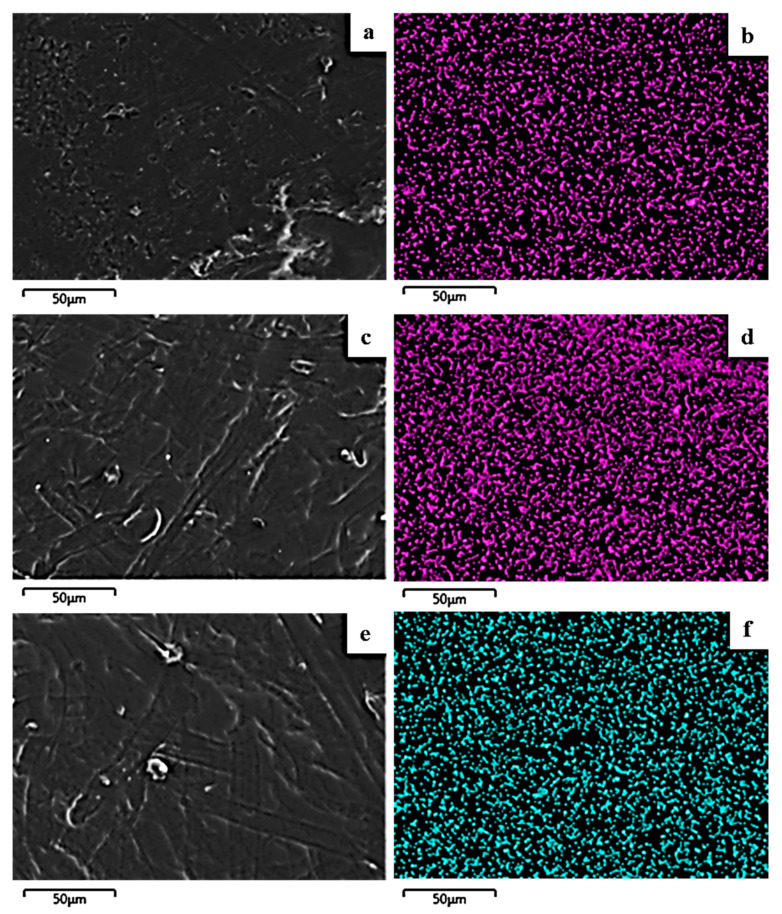
Secondary electron SEM images and respective EDS compositional mapping of silicon for the films: (**a**,**b**) PLLA/1%TO@OrgMt, (**c**,**d**) PLLA/3%TO@OrgMt, and (**e**,**f**) PLLA/5%TO@OrgMt.

Figure 9 shows the surface (b,d,f) and cross-section (a,c,e) topology of the PLLA/1TO@OrgMt, PLLA/3TO@OrgMt, and PLLA/5TO@OrgMt nanocomposites, indicating significant clay dispersion within the PLLA polymer matrix. In contrast to the TO@NaMt nanocomposites, the disperse of the TO@OrgMt clays in the polymer matrix led to nonaggregated distributions. This is indicated by the Si signal through EDS mapping. The SEM images and EDS mapping analysis of the PLLA/xTO@OrgMt materials confirm the enhanced compatibility of the TO@OrgMt clay with the PLLA polymer matrix compared to the compatibility of the TO@NaMt clay, which led to rougher PLLA/xTO@NaMt surfaces.

The results from SEM/EDS measurements are in accordance with the XRD results where an intercalated nanocomposite structure for TO@OrgMt-based films and an exfoliated nanocomposite structure for TO@NaMt-based films was confirmed. Both XRD and SEM/EDS results are in total agreement with the higher tensile and thermal strength, which was observed for TO@OrgMt-based films compared to the respective properties of the TO@NaMt-based films.

### 2.6. Barrier Properties

Table 3 lists the measured Water Vapor Transmission Rate (*WVTR*) and Oxygen Transmission Rate (OTR) values as well as the calculated water vapor diffusion coefficient (*D_WV_*) and oxygen permeability coefficient (*Pe_O2_*) values. Water vapor diffusion coefficient values (*D_WV_*) and oxygen permeability coefficient values (*Pe_O2_*) were calculated according to Fick law, oxygen permeability through polymers equation, and assumptions described in [28]. According to this literature, we can calculate the water vapor diffusion coefficient D (cm^2^·s^−1^) for every film as follows:

(1) $$D_{WV}=WVTR\times \frac{\Delta x}{\Delta C}$$

where *WVTR* [G·cm^−2^·s^−1^)] is the water vapor transmission rate, Δ*x* (cm) is the film thickness, and Δ*C* (g·cm^−3^) is the humidity concentration gradient in the two opposite sides of the film. Furthermore, we can calculate oxygen permeability through films as follows:

(2) $$\frac{J}{A}=Pe_{O2}\times \frac{\Delta C}{\Delta x}$$

where *J/A* (mol·cm^−2^·s^−1^) is the specific amount of gas pass through the membrane, *Pe*_*O*2_ (cm^2^·s^−1^) is the permeability coefficient, Δ*C* (mol·cm^−3^ STP) is the pressure gradient in the two opposite sides of the membrane, and Δ*x* (cm) is the membrane thickness.

Rearranging Equation (1), we can express the permeability coefficient as follows:

(3) $$Pe_{O2}=\frac{J}{A\times \Delta C}\times \Delta x$$

Based on these two mass transfer parameters and considering the value of 5 wt% as the maximum additive concentration in the PLLA matrix, it is obvious from Table 3 that the addition of NaMt clay in the PLLA matrix decreases the water vapor barrier, while the addition of OrgMt clay increases this property. The presence of TO in both types of clay increases the water vapor barrier further. In contrast, the oxygen barrier increases by the addition of either NaMt or OrgMt in the PLLA matrix. Similarly to the water vapor diffusion case, the addition of TO in both types of clay leads to higher oxygen barrier values. In conclusion, we can say that the exfoliated PLLA/NaMt samples exhibit lower water vapor barrier values, even from values of the pure PLLA, compared to the intercalated PLLA/OrgMt samples, which exhibit higher water vapor barrier values from the values of both the pure PLLA and the PLLA/NaMt samples. In all cases, the oxygen permeability barrier increases.

### 2.7. Antioxidant Activity

Table 3 reports the antioxidant activity values calculated from Equation (4). All tested films exhibit significant antioxidant activity, which indicates that, potentially, they could be used as active packaging films. It is obvious from measurements that the antioxidant activity increases as the content of TO@NaMt and TO@OrgMt nanostructure increases. Antioxidant activity was calculated by absorbance measurements of DPPH ethanolic solution at 517 nm. The presence of film pieces in such solutions decreases the absorbance values. The following equation estimates the % antioxidant activity of each sample film:

% Antioxidant activity = (Abs_control_ − Abs_sample_)/Abs_control_ × 100 (4)

where Abs_control_ is the absorbance value of pure DPPH ethanolic solution and Abs_sample_ is absorbance value of DPPH ethanolic solution after incubation with 30 mg sample film for 24 h in a dark place and at 25 °C. TO@OrgMt-based films exhibit higher antioxidant activity compared to that of the TO@NaMt-based films. This result could be attributed to the higher TO content of TO@OrgMt nanostructure (36.2 wt%) over that of TO@NaMt nanostructure (22.0 wt%).

### 2.8. Antimicrobial Properties

Antibacterial activity of thyme oil and PLLA films was tested against four food pathogenic bacteria, *E. coli*, *S. aureus*, *S. enterica*, and *L. monocytogenes*. The inhibition zones indicated strong antimicrobial activity of pure thyme oil against the tested bacteria. Thyme oil totally inhibited the *E. coli* and *L. monocytogenes* and also exhibited good antibacterial activity against *S. aureus* and *S. enterica.* The mean inhibition zone diameter for the last two microorganisms was 55 mm and 27 mm, respectively. On the contrary, the tested films did not show antibacterial effect neither in terms of surrounding clear zone nor underneath the film. Many parameters could affect the film’s antimicrobial properties, e.g., the nature of the EO, the quantity or the mass ratio of EO into the polymer, and maybe the combination of some of them. By reviewing the results, it seems that the film formation process by hot-pressing at high temperatures (160 °C) could evaporate the EO’s volatile and bioactive compounds, leading to a high decrease of their antibacterial activity. Additionally, this hot-pressing process probably removes the EO from the tested film and reduces drastically the EO’s concentration in films, causing a non-antimicrobial effect. Finally, it was reported in the literature the possibility that the controlled EO’s releasing from the polymer films could limit the diffusion of the EO’s active components [29].

## 3. Materials and Methods

### 3.1. Materials

#### 3.1.1. Used Essential Oil

Thyme oil used was purchased from Esperis S.p.a., Binda, Milano, Italy, and, according to safety data sheets, consists of 50–60% thymol; 15–20% para-cymene; 10–12.5% 1-isopropyl-4-methyl-1,4-cyclohexadiene p-mentha-1,4-diene; 3–5% carvacrol; and 1–3% linalool, beta-caryophyllene, beta-myrcene, (R)-p-mentha-1,8-diene, alpha-pinene, borneol, and terpinene-4-olo.

#### 3.1.2. Used Clay

(i) Sodium exchanged montmorillonite (NaMt) with code name Nanomer^®^ PGV with mass density 2.6 g/cm^3^ and CEC value 145 meq/100 g was produced by Nanocor Company (Hoffman Estates, IL, USA) and supplied by Aldrich (St. Louis, MO, USA). The chemical composition of NaMt was 62.9% SiO_2_, 19.6% Al_2_O_3_, 3.35% Fe_2_O_3_, 3.05% MgO, 1.68% CaO, and 1.53% Na_2_O. (ii) Organo-montmorillonite (OrgMt) NANOMER^®^-I·44P was produced by Nanocor Company (Hoffman Estates, IL, USA) and supplied by Aldrich (St. Louis, MO, USA). NANOMER^®^-I.44P is an ammonium ion-modified clay containing ~40 wt% dimethyl dialkyl (C14-18) ammonium organic modifier.

#### 3.1.3. Used PLLA

PLLA was supplied by NatureWorks, Minnetonka, MN, USA, with the trade name Ingeo™ Biopolymer 3052D.

### 3.2. Methods

#### 3.2.1. Preparation of TO@NaMt and TO@OrgMt Nanostructures

The encapsulation of TO in both NaMt and OrgMt clays was performed via a green evaporation/adsorption process described in detail previously [20]. The final TO loading on obtained TO@NaMt and TO@OrgMt nanostructures was calculated gravimetrically and was approx. 22.0 wt% and 36.2 wt%, respectively.

#### 3.2.2. Preparation of PLLA/TO@NaMt and PLLA/TO@OrgMt Films

The PLLA/TO@NaMt and PLLA/TO@OrgMt films were prepared via a melt-mixing process. For the preparation, a minilab co-rotating twin extruder (Haake Mini Lab II, ThermoScientific, ANTISEL, S.A., Athens, Greece) was used. The uniform operating temperature was 170 °C at a screw speed of 100 rpm for 5 min total processing time. The nominal compositions of TO@NaMt and TO@OrgMt nanohybrids added to PLLA were fixed to 1 wt%, 3 wt%, and 5 wt%. “Blank” samples were also prepared for comparison by mixing PLLA with commercial NaMt and OrgMt with the same nominal compositions. The obtained melt compound strands were cut into small granules using a granulating machine. Finally, films were produced with approx. 10 cm diameter by hot-pressing of approximately 2 g of the obtained granules at 160 °C under 3.0 megapascal (MPa) constant pressure for 3 min using a hydraulic press with heated platens.

### 3.3. XRD Analysis

The crystalline structure of all PLLA/NaMt, PLLA/TO@NaMt, PLLA/OrgMt, and PLLA/TO@OrgMt films was studied via XRD analysis measurements using a Brüker D8 Advance X-ray diffractometer (Brüker, Analytical Instruments, S.A. Athens, Greece) equipped with a LINXEYE XE High-Resolution Energy-Dispersive detector.

### 3.4. FTIR Spectrometry

The chemical structure and the possible interactions of PLLA chains with NaMt, OrgMt, TO@NaMt, and TO@OrgMt nanostructures of all PLLA/NaMt, PLLA/TO@NaMt, PLLA/OrgMt, and PLLA/TO@OrgMt films were studied by IR spectra measurements. The obtained infrared (FTIR) plots were the average of 32 scans at 2 cm^−1^ resolution and were measured using an FT/IR-6000 JASCO Fourier transform spectrometer (JASCO, Interlab, S.A., Athens, Greece) in the wavenumber range of 4000–400 cm^−1^.

### 3.5. Thermal Studies TG–DTA

Thermogravimetric (TGA) and differential thermal analysis (DTA) were carried out on all obtained films. Measurements were performed using a PerkinElmer Pyris Diamond TGA/DTA instrument (Interlab, S.A., Athens, Greece). Samples of approximately 5 mg were heated under N_2_ flow from 25 to 700 °C at a rate of 5 °C/min.

### 3.6. Tensile Properties

Tensile measurements were carried out on all prepared films according to the ASTM D638 method. A Simantzü AX-G 5 kNt instrument (Simantzu. Asteriadis, S.A., Athens, Greece) was used. Three to five samples of each film were tensioned at an across head speed of 2 mm/min. The samples were dumbbell-shaped with gauge dimensions of 10 mm × 3 mm × 0.22 mm. Force (N) and deformation (mm) were recorded during the test.

### 3.7. Scanning Electron Microscopy (SEM)/Energy Dispersive Spectroscopy (EDS)

Scanning electron microscopy (SEM) images were obtained using a JEOL JSM-6510 LV SEM Microscope (JEOL Ltd., Tokyo, Japan) equipped with an X-Act EDS detector by Oxford Instruments, Abingdon, Oxfordshire, UK (an acceleration voltage of 20 kV was applied) for elemental and mapping analysis. The specimens were sputtered with an Au-Pd thin film (4–8 nm) using a mini sputter coater SC7620 from Quorum Technologies LTD (Kent, UK).

### 3.8. Water Vapor Transmission Rate (WVTR)

WVTR of all obtained films was determined. Experimental conditions were fixed at 38 °C and 50% RH according to the ASTM E96/E96M-05 method. Using a handmade apparatus and following the methodology described extensively in a previous publication [30] and modified recently [22,28], WVTR measurements were carried out. For such measurements, film disks of 2.5 cm diameter and 100 μm thickness were used.

### 3.9. Oxygen Permeability (OP)

The oxygen transition rate (OTR) of all obtained films was analyzed using an oxygen permeation analyzer (8001, Systech Illinois Instruments Co., Johnsburg, IL, USA). All samples were tested at 23 °C and 0% RH according to the ASTM D 3985 method. OTR values were expressed at cc O_2_/m^2^/day. The average film thickness of the tested samples was in the range of 350 to 400 μm. The mean OTR value for each kind of film resulted from the measurements of three samples.

### 3.10. Antioxidant Activity

The antioxidant activity of films was evaluated using 300 mg of small pieces (approximately 3 mm × 3 mm) of each film. The sample was placed in a dark-colored glass bottle with a plastic screw cap and filled with 10 mL DPPH ethanolic solution of 50 ppm (mg/L) concentration. After incubation at 25 °C for 24 h in darkness, the % antioxidant activity values of the films were calculated according to Equation (4). Antioxidant measurements were carried out using a JASCO V530 UV-VIS instrument (JASCO International CO., Ltd., Tokyo, Japan).

### 3.11. Antimicrobial Assay

PLLA films were tested by agar diffusion method for their efficacy against Gram-negative bacteria *Escherichia coli* (ATCC 25922) and *Salmonella enterica* subsp. *enterica* (DSMZ 17420) and Gram-positive bacteria *Staphylococcus aureus* (DSMZ 12463) and *Listeria monocytogenes* (DSMZ 27575). The above bacteria isolates were provided from the Institute of Technology of Agricultural Products, ELGO-DEMETER, Lykovryssi, Greece.

The stock bacteria colonies were cultured overnight in Mueller–Hinton broth to obtain a range of 10^7^–10^8^ CFU mL^−1^. Then, a sterile cotton swab was used to inoculate the surface of Mueller–Hinton agar dishes. To achieve homogeneous growth, the plates were rotated every 60° during the inoculation process.

Films were cut into 6 mm discs by a circular knife and sterilized using a UV lamp. Film discs were placed on the surface of Mueller–Hinton agar plates, and the diameter of inhibitory zones was measured after 24 h of incubation at 37 °C. Moreover, the growth of bacteria on the contact area between the films and the agar surface was also evaluated. Thyme oil antibacterial activity was examined by the agar well diffusion method. The EO was cast into Mueller–Hinton agar wells with 6 mm diameter, and the clear zone of inhibition was recorded. The experiment was repeated three times.

### 3.12. Statistical Analysis

According to the literature, it is usual in such studies to measure every property at least three times under the same conditions to achieve accurate mean values standard deviation values and standard error. Moreover, it is usual to test the inequality of mean values between all kind of samples to promote the better material which exhibits superior properties. All the experimental data for E, σ_uts_, ε_b_ (%), WVTR, % water sorption, OTR, *E. coli*, *S. aureus*, *S. enterica*, and *L. monocytogenes* were statistically treated using the statistical software SPSS ver. 20. Statistical tests were carried the way described in previous literature reports [28].

## 4. Conclusions

Characterization techniques indicated that the interplay and compatibility between PLLA chains and clay platelets was more intense in the case of the intercalated OrgMt clay compared to that of the partially exfoliated NaMt clay. This also means a better mixing of these two materials, i.e., PLLA and OrgMt. Furthermore, in both cases of clay addition, the presence of TO boosted the mixing process. The thermal stability of the PLLA matrix was improved by addition of either NaMt or OrgMt clay. In the case of the OrgMt clay, the increase of such stability was higher. Nevertheless, there was an optimum addition around 3 wt% for optimum mechanical properties. The exfoliated PLLA/NaMt samples exhibited lower water vapor barrier values, even from values of the pure PLLA, compared to the intercalated PLLA/OrgMt samples, which exhibited higher water vapor barrier values from the values of both the pure PLLA and the PLLA/NaMt samples. In all cases, the oxygen barrier increased, and the TO addition decreased, water vapor diffusion and oxygen permeability. Higher TO adsorption capacity of OrgMt-based materials led to higher antioxidant activity. As an overall validation of the materials in our study, all samples could be potentially used for active food packaging applications, but the PLLA/5%TO@OrgMt film seems to be the most advantageous.

## Figures and Tables

**Table 1:** Code names and amounts in grams used for the preparation of pure PLLA film and all obtained PLLA/NaMt, PLLA/TO@NaMt, PLLA/OrgMt, and PLLA/TO@OrgMt nanocomposite films.

Code Name	PLLA	NaMt	OrgMt	TO@NaMt	TO@OrgMt
PLLA	5	-	-	-	-
PLLA/1NaMt	4.95	0.05			
PLLA/3NaMt	4.85	0.15	-	-	-
PLLA/5NaMt	4.75	0.25			
PLLA/1TO@NaMt	4.95			0.05	
PLLA/3TO@NaMt	4.85	-	-	0.15	-
PLLA/5TO@NaMt	4.75	-	-	0.25	-
PLLA/1OrgMt	4.95		0.05		
PLLA/3OrgMt	4.85	-	0.15	-	-
PLLA/5OrgMt	4.75		0.25		
PLLA/1TO@OrgMt	4.95				0.05
PLLA/3TO@OrgMt	4.85	-	-	-	0.15
PLLA/5TO@OrgMt	4.75	-	-	-	0.25

**Table 2:** Mean values (Standard Deviation) of modulus of elasticity (E), tensile strength (σ_uts_), and % elongation at break (ε_b_) of all tested PLLA/NaMt, PLLA/TO@NaMt, PLLA/OrgMt, and PLLA/TO@OrgMt films.

Code Name	Tensile E (St. Dev.) (MPa)	σ_uts_ (MPa) (St. Dev.)	ε_b_ (%) (St. Dev.)
PLLA	2891.3 (61.9)	33.9 (4.9)	1.4 (0.2)
PLLA/1NaMt	2460.2 (232.3)	31.7 (5.4)	1.3 (0.2)
PLLA/3NaMt	2660.5 (137.8)	32.5 (6.0)	1.3 (0.3)
PLLA/5NaMt	1823.3 (285.4)	22.0 (6.3)	1.1 (0.1)
PLLA/1TO@NaMt	2609.5 (137.8)	32.2 (6.0)	1.4 (0.1)
PLLA/3TO@NaMt	2910.7 (268.3)	34.5 (2.1)	1.4 (0.1)
PLLA/5TO@NaMt	2811.4 (121.7)	33.5 (3.7)	1.5 (0.3)
PLLA/1OrgMt	2315.5 (185.2)	29.9 (3.8)	1.8 (0.3)
PLLA/3OrgMt	2965 (210.4)	34.2 (6.2)	1.5 (0.2)
PLLA/5OrgMt	2483 (235.2)	22.5 (5.3)	1.0 (0.2)
PLLA/1TO@OrgMt	2971 (145.8)	38.7 (4.2)	1.9 (0.3)
PLLA/3TO@OrgMt	3277 (181.6)	40.3 (4.8)	1.9 (0.1)
PLLA/5TO@OrgMt	2758.5 (206.7)	18.2 (5.3)	0.9 (0.2)

**Table 3:** Water Vapor Transmission Rate (WVTR), Oxygen Transmission Rate (OTR), and antioxidant activity values of all tested PLLA/xclay and PLLA/xTO@clay composite films.

Sample Code Name	WVTR (g·m^−2^·day^−1^)	D_WV_ (cm^2^·s^−1^)	OTR (cm^3^·m^−2^·day^−1^)	Pe_O2_ (cm^2^·s^−1^)	Antioxidant Activity after 24 h
PLLA	15.9 ± 1.3	9.64 (±0.9) × 10^−12^	12.4 ± 0.9	5.39 (±0.6) × 10^−10^	n.d. ^1^
PLLA/1NaMt	16.1 ± 1.2	9.76 (±0.8) × 10^−12^	9.8 ± 0.8	4.26 (±0.5) × 10^−10^	n.d.
PLLA/3NaMt	16.4 ± 1.4	9.96 (±1.1) × 10^−12^	9.4 ± 0.8	4.06 (±0.5) × 10^−10^	n.d.
PLLA/5NaMt	16.7 ± 1.0	10.14 (±0.7) × 10^−12^	9.2 ± 0.7	4.00 (±0.4) × 10^−10^	n.d.
PLLA/1TO@NaMt	15.0 ± 1.1	9.11 (±0.8) × 10^−12^	9.0 ± 0.6	3.91 (±0.3) × 10^−10^	11.9 ± 1.2
PLLA/3TO@NaMt	15.1 ± 1.2	9.17 (±0.8) × 10^−12^	8.9 ± 0.6	3.88 (±0.3) × 10^−10^	19.7 ± 1.3
PLLA/5TO@NaMt	15.5 ± 1.3	9.41 (±0.9) × 10^−12^	8.8 ± 0.7	3.84 (±0.4) × 10^−10^	24.1 ± 1.5
PLLA/1OrgMt	14.9 ± 0.9	9.03 (±0.6) × 10^−12^	9.3 ± 0.8	4.03 (±0.5) × 10^−10^	n.d.
PLLA/3OrgMt	14.1 ± 0.9	8.54 (±0.6) × 10^−12^	8.4 ± 0.7	3.65 (±0.4) × 10^−10^	n.d.
PLLA/5OrgMt	13.6 ± 0.8	8.26 (±0.5) × 10^−12^	6.3 ± 0.5	2.72 (±0.2) × 10^−10^	n.d.
PLLA/1TO@OrgMt	14.0 ± 1.0	8.50 (±0.7) × 10^−12^	9.1 ± 0.6	3.95 (±0.3) × 10^−10^	17.2 ± 1.8
PLLA/3TO@OrgMt	13.6 ± 0.8	8.26 (±0.5) × 10^−12^	8.0 ± 0.6	3.48 (±0.3) × 10^−10^	38.0 ± 2.1
PLLA/5TO@OrgMt	13.0 ± 0.7	7.92 (±0.4) × 10^−12^	5.3 ± 0.5	2.30 (±0.2) × 10^−10^	50.3 ± 2.3

^1^ n.d. (not detected).

## Data Availability

The datasets generated for this study are available on request to the corresponding author.

## Sample Availability

Samples of the compounds are available from the authors.
